# Complete Genome Sequence of Ralstonia solanacearum WR-1, a Virulent Strain on Solanum lycopersicum

**DOI:** 10.1128/mra.01345-22

**Published:** 2023-05-01

**Authors:** HwangWeon Jeong, Jae-Hyeon Oh, Sung-Mi Kim, Young-Joo Seol, Hye-Jin Yoon, Jinjeong Lee, Su Jung Ra, Eunhee Kim, Myeong Jin Kang, Il-Pyung Ahn

**Affiliations:** a National Institute of Agricultural Sciences, Jeonju, Korea; University of Arizona

## Abstract

Ralstonia solanacearum is a bacterial wilt pathogen of Solanum lycopersicum. Its pathogenicity is the result of coevolution during continuous interaction with its host plants under given biotic and abiotic environments. To elucidate clues for pathogenicity of our WR-1 strain, its genome sequence was analyzed.

## ANNOUNCEMENT

Ralstonia solanacearum is a core species of the R. solanacearum species complex (RSSC) that is a notorious bacterial wilt pathogen in economically important crops, such as tomato (1, 2). The RSSC is a heterogeneous assemblage of related but genetically and/or phenotypically distinct populations (3). Even more, RSSC harbors an additional two species (Ralstonia pseudosolanacearum and Ralstonia syzygii) and several subspecific groups including races. Races are determined by host spectrum, that is, the pathogenicity of RSSC strains is the criterion of differentiation (4). To invade its host plants, bacterial wilt pathogen RSSC members evolved effectors disturbing rapid and efficient host defense signaling, governed by resistance gene products sensing and systemically disseminating a pathogen infection. The pathogen also equipped finely tuned apparatus paving the way for delivering virulence factors like effectors directly from the pathogen’s cytoplasm to the target host cells (5, 6).

Strain WR-1 was retrieved from vascular tissues of tomato plants heavily infected by R. solanacearum in Korea and preserved at the Korean Agricultural Culture Collection (KACC number 10695). A single colony of WR-1 was streaked onto casamino acid-peptone-glucose agar and grown at 28°C for 48 h. The propagated cells were harvested from the plate for DNA preparation (Pacific Biosciences, CA). DNA quality was good enough for subsequent work (Femto Pulse; Agilent, CA), and a Sequel library was constructed according to the 15-kbp SMRTbell template library workflow (Pacific Biosciences). The sequence of the library was analyzed on a Sequel sequencing system (Pacific Biosciences). In total, there were 100,091 subreads and 882,335,361 bases. The *N*_50_ value was 10,817. *De novo* assembly using the microbial assembly application in the PacBio_SMRT program resulted in two circular contigs. A TruSeq library was constructed (TruSeq nano DNA high-throughput library prep kit; Illumina, CA, USA), and the sequence was analyzed on a HiSeq-x instrument. The number of TruSeq total reads was 12,599,011, and the Q30 was 97.67%.

Two contigs generated from Sequel subreads, namely, a chromosome and a megaplasmid, were 3,893,306 bp and 1,966,100 bp, respectively, and their GC contents were 66.7% and 67.2%, respectively. The read depths were 117.9× and 171.7×. Mapping HiSeq reads using Pilon validated two Sequel assemblies perfectly with 311.46× read depth. Annotation by the Prokaryotic Genome Annotation Pipeline indicated 3,514 coding DNA sequences (CDSs), 60 tRNAs, and 9 rRNAS on the chromosome and 1,528 CDSs, 6 tRNAs, and 3 rRNAs on the megaplasmid. The geographic origin of the type strain GMI1000 is French Guiana on the northern Atlantic coast of South America. To analyze strain-specific variation between GMI1000 and WR-1 in respect to predicted pathogenicity, which is affected largely by the given biotic and abiotic environmental conditions, genes of WR-1 that participate in the protein secretion system and encode type 3 effectors were analyzed using TXSScan (7) and Ralsto T3E (8). WR-1 has 186 genes involved in the type 1 secretion system (T1SS; 33 genes), T2SS (27 genes), T3SS (15 genes), T4SS (30 genes), T5SS (16 genes), and T6SS (31 genes) and encoding flagella/pili (17/18 genes). Similarly, GMI1000 strains contain 192 correspondences. Our WR-1 has 57 genes encoding type 3 effectors and those of GMI1000 is 71.

### Data availability.

The genome information of the chromosome and megaplasmid of WR-1 was deposited in GenBank with accession numbers CP102184 and CP102185. Raw sequences of TruSeq and Sequel reads were submitted to NCBI SRA and the accession numbers are SRR20115430 and SRR20115431 under BioProject number PRJNA863051 and BioSample number SAMN29673693.
